# Supplementary material for: TERRA increases at short telomeres in yeast survivors and regulates survivor associated senescence (SAS)
Source: Nucleic Acids Res. 2022 Dec 14;50(22):12829–43. doi: 10.1093/nar/gkac1125 (PMC9825167; doi:10.1093/nar/gkac1125)
Supplement: gkac1125_Supplemental_File [file gkac1125_supplemental_file.pdf]

Supplementary Material

TERRA increases at short telomeres in yeast survivors and regulates survivor associated senescence (SAS)

Stefano Misino, Anke Busch, Carolin B. Wagner, Fabio Bento and Brian Luke

Supplemental Data (Figures S1 – S4)

Supplemental Tables (Tables S1 – S3)

Supplementary Data

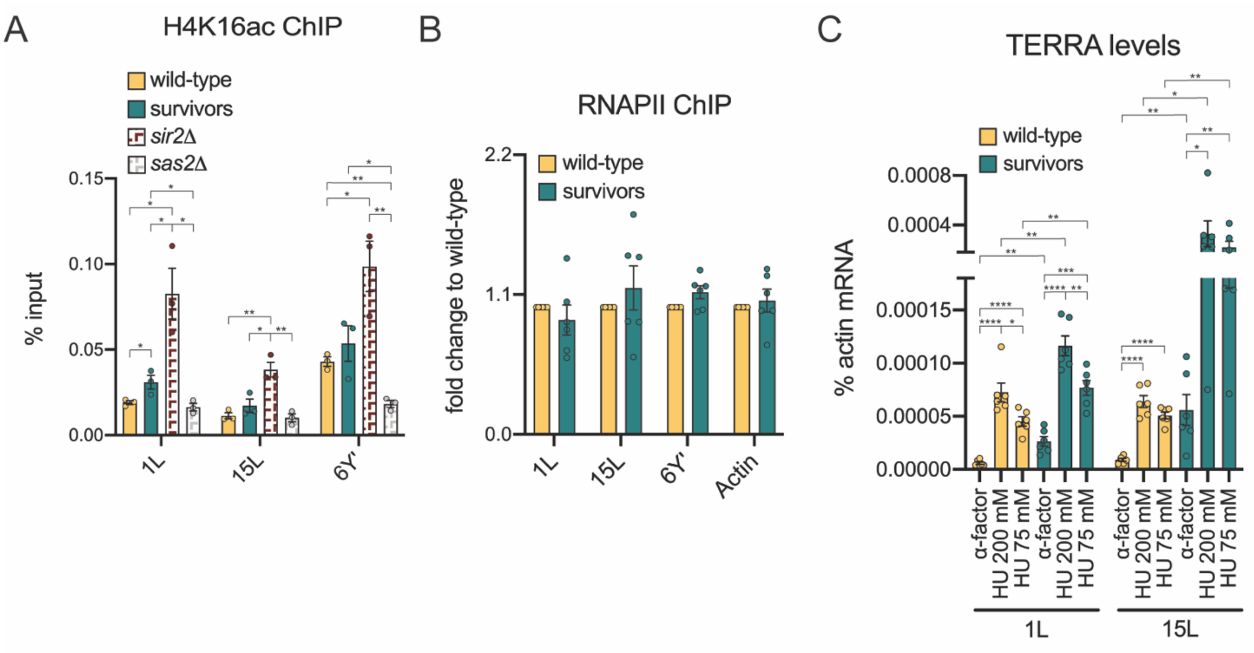

Figure S1. Survivors accumulate TERRA RNA-DNA hybrids and uphold TERRA cell cycle regulation

(A) Acetylation of histone H4 lysine 16 was quantified by ChIP at 1L, 15L and 6Y' telomeres in wild-type, survivor, *sir2Δ* and *sas2Δ* cells. The last two strains were used as positive and negative control, respectively. The IP values were normalized by the input, resulting in "% input" shown in the graph.

Mean + SEM is displayed, n=3. p values were calculated by an unpaired two-tailed Student's *t*-test (*p<0.05, **p<0.01).

(B) ChIP of RNA pol II at 1L, 15L and 6Y' telomeres in wild-type and survivor cells. The "% input" values of all samples were normalized by the ones of wild-type cells to obtain the final "fold change to wild-type". The actin locus was used as a positive control.

(C) TERRA levels were measured at telomere 1L and 15L in *tlc1Δ* survivors and wild-type cells arrested in G1 (α-factor), early S-phase (HU 200 mM) and late S-phase (HU 75 mM). The amount of TERRA is expressed as "% actin mRNA". HU indicates hydroxyurea.

Mean + SEM is displayed, n=6. p values were calculated by an unpaired two-tailed Student's *t*-test (*p<0.05, **p<0.01, ***p<0.001, ****p<0.0001).

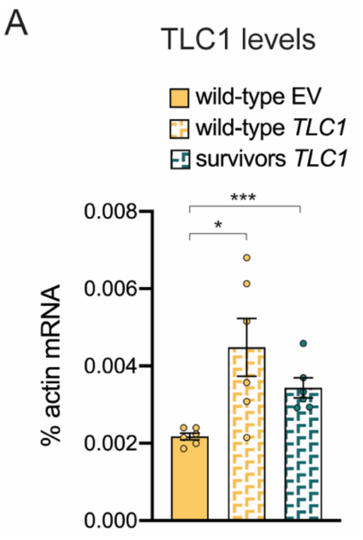

Figure S2. TLC1 expression in wild-type and survivor cells
(A) TLC1 expression levels were monitored in wild-type cells transformed with an empty vector (EV) and in wild-type cells and *tlc1Δ* survivors transformed with a vector expressing the gene under its endogenous promoter (*TLC1*). The expression levels are represented as "% actin mRNA".
Mean + SEM is displayed, n=6. p values were calculated by an unpaired two-tailed Student's *t*-test (*p<0.05, ***p<0.001).

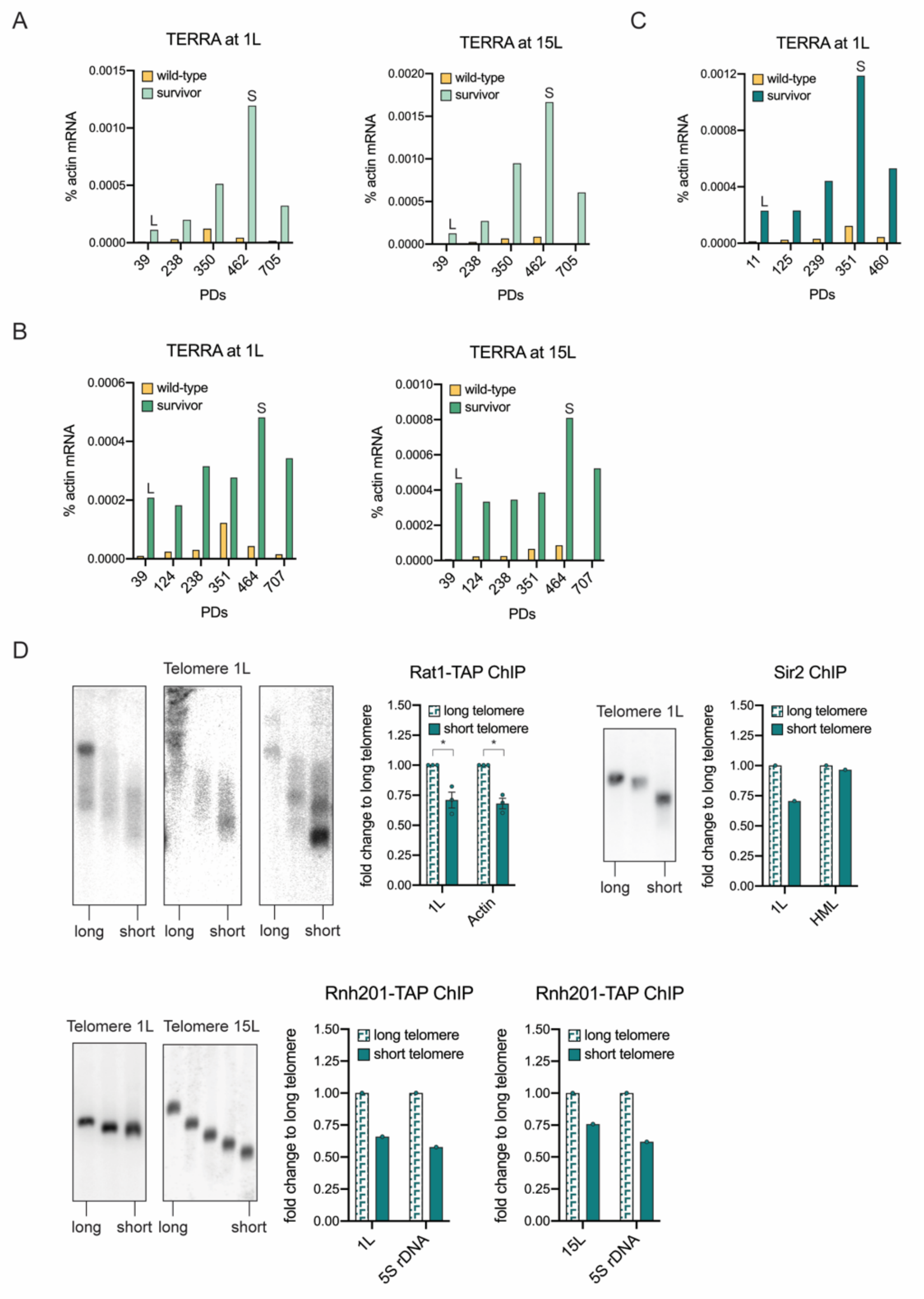

Figure S3. TERRA levels increase at short telomeres in survivors
(A, B, C) TERRA levels were monitored in 3 independent survivor clones at telomere 1L and 15L. "L" and "S" indicate respectively when telomeres were long and when they shortened as followed

by Southern blots. The levels of TERRA deriving from these telomeres are plotted in Figure 3F. 15L TERRA levels are not reported for clone C as that telomere underwent an early recombination event and acquired a Y' element, hence complicating interpretation of the result.

(D) Rat1, Sir2 and Rnh201 binding to telomere 1L and 15L were monitored in independent survivor clones as telomeres shortened. Southern blots of the respective telomeres are shown indicating the length, "long" or "short", at which the proteins enrichment was measured. In case of Rat1-TAP and Rnh201-TAP ChIPs, the experiment was performed for both the strains harboring the tagged or untagged version of the protein. The "% input" values of TAP-tagged survivors were divided by the ones of the untagged equivalents and the resulting fold change was divided by the one corresponding to the "long" telomere. This "fold change to long telomere" is plotted. The actin, HML and 5S rDNA loci were used as positive controls.

For Rat1-TAP ChIP, mean + SEM is displayed, n=3. p values were calculated by an unpaired two-tailed Student's *t*-test with Welch's correction (*$p<0.05$).

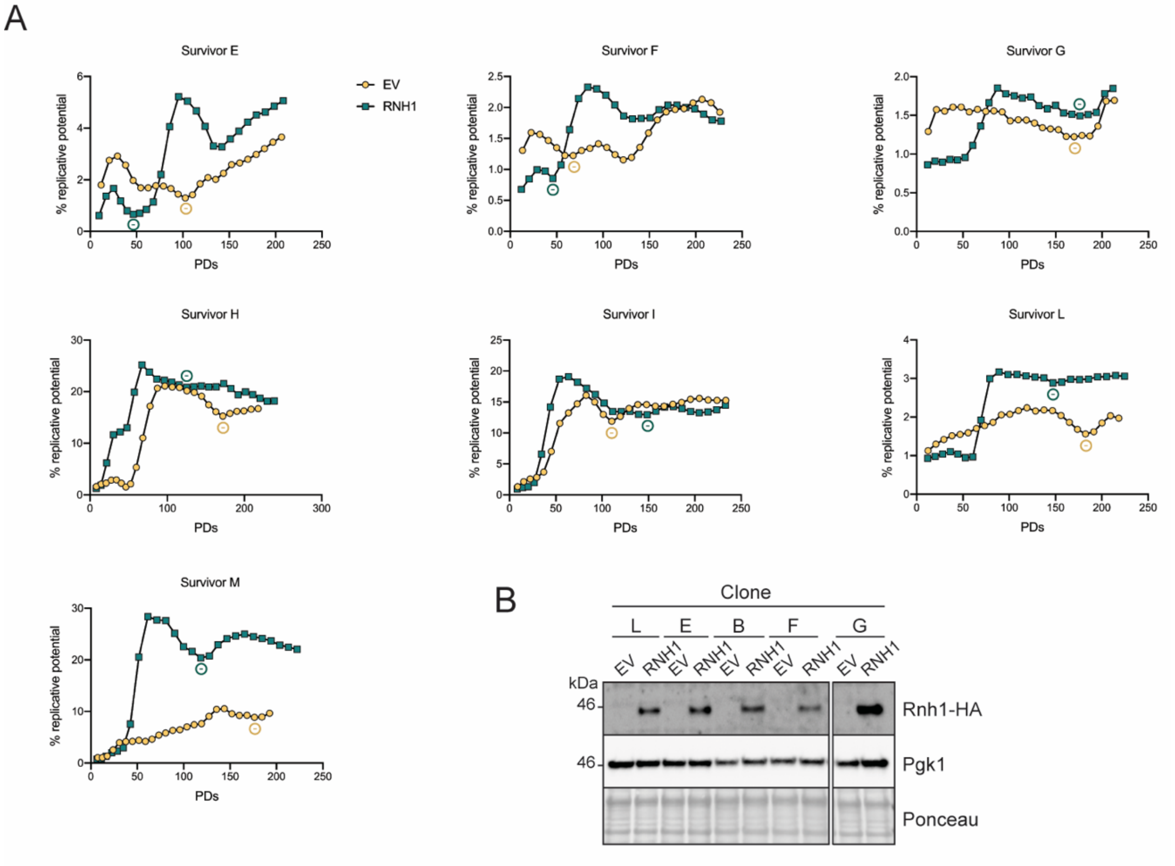

Figure S4. SAS rates are increased with RNase H1 overexpression

(A) All growth curves contributing to the data in Figure 4E and obtained in a manner similar to Figures 4A and 4B.

(B) Western blot showing the overexpression of Rnh1-HA in survivors from Figure 4A. Pgk1 and Ponceau staining were used as loading control. These extracts were performed on the last day of the passaging experiment.

Supplementary Tables

Supplementary Table S1: Yeast strains used in this study

Name	Features	Description
yDB13	Wild-type A	*MATa; his3Δ1; leu2Δ0; ura3Δ0; met15Δ0; bar1::KAN + pBL189-URA3*
yDB15	Wild-type B	*MATa; his3Δ1; leu2Δ0; ura3Δ0; met15Δ0; bar1::KAN + pBL189-URA3*
yDB05	Survivor A	*MATa; his3Δ1; leu2Δ0; ura3Δ0; met15Δ0; bar1::KAN; tlc1::HIS3*
yDB06	Survivor B	*MATa; his3Δ1; leu2Δ0; ura3Δ0; met15Δ0; bar1::KAN; tlc1::HIS3*
yDB07	Survivor C	*MATa; his3Δ1; leu2Δ0; ura3Δ0; met15Δ0; bar1::KAN; tlc1::HIS3*
yDB08	Survivor D	*MATa; his3Δ1; leu2Δ0; ura3Δ0; met15Δ0; bar1::KAN; tlc1::HIS3*
ySM269	Wild-type (Rat1 no-TAP)	*MATa; his3Δ1; leu2Δ0; ura3Δ0; met15Δ0*
ySM270	Wild-type (Rat1 no-TAP)	*MATa; his3Δ1; leu2Δ0; ura3Δ0; met15Δ0*
yBL7	Wild-type	*MATa; his3Δ1; leu2Δ0; ura3Δ0; met15Δ0*
ySM 272	Wild-type (Rat1-TAP)	*MATα; his3Δ1; leu2Δ0; ura3Δ0; met15Δ0; rat1-TAP::HIS3*
ySM273	Wild-type (Rat1-TAP)	*MATα; his3Δ1; leu2Δ0; ura3Δ0; met15Δ0; rat1-TAP::HIS3*
ySM281	Survivor (Rat1 no-TAP)	*MATα; his3Δ1; leu2Δ0; ura3Δ0; met15Δ0; tlc1::NAT*
ySM282	Survivor (Rat1 no-TAP)	*MATa; his3Δ1; leu2Δ0; ura3Δ0; met15Δ0; tlc1::NAT*
ySM283	Survivor (Rat1 no-TAP)	*MATa; his3Δ1; leu2Δ0; ura3Δ0; met15Δ0; tlc1::NAT*
ySM284	Survivor (Rat1-TAP)	*MATα; his3Δ1; leu2Δ0; ura3Δ0; met15Δ0; tlc1::NAT; rat1-TAP::HIS3*
ySM285	Survivor (Rat1-TAP)	*MATα; his3Δ1; leu2Δ0; ura3Δ0; met15Δ0; tlc1::NAT; rat1-TAP::HIS3*
ySM286	Survivor (Rat1-TAP)	*MATα; his3Δ1; leu2Δ0; ura3Δ0; met15Δ0; tlc1::NAT; rat1-TAP::HIS3*
ySM321	Survivor (Rnh201 no-TAP)	*his3Δ1; leu2Δ0; ura3Δ0; met15Δ0; est2::NAT*
ySM322	Survivor (Rnh201 no-TAP)	*his3Δ1; leu2Δ0; ura3Δ0; met15Δ0; est2::NAT*

ySM326	Survivor (Rnh201-TAP)	*his3Δ1; leu2Δ0; ura3Δ0; met15Δ0; est2::NAT; rnh201-TAP::HIS*
ySM329	Survivor (Rnh201-TAP)	*his3Δ1; leu2Δ0; ura3Δ0; met15Δ0; est2::NAT; rnh201-TAP::HIS*
ySM338	Wild-type (Rnh201-TAP)	*his3Δ1; leu2Δ0; ura3Δ0; met15Δ0; rnh201-TAP::HIS*
ySM342	Wild-type (Rnh201-TAP)	*his3Δ1; leu2Δ0; ura3Δ0; met15Δ0; rnh201-TAP::HIS*
ySM345	Wild-type (Rnh201 no-TAP)	*his3Δ1; leu2Δ0; ura3Δ0; met15Δ0;*
ySM346	Wild-type (Rnh201 no-TAP)	*his3Δ1; leu2Δ0; ura3Δ0; met15Δ0;*
ySM81-86	Survivor to be transformed with pBL211 (EV) or pBL352 (RNH1)	*MATa; his3Δ1; leu2Δ0; ura3Δ0; met15Δ0; tlc1::HIS*
yVP345	Survivor (*rnh201Δ*) to be transformed with pBL97 (EV) or pBL401 (RNH201)	*MATα; his3Δ1; leu2Δ0; ura3Δ0; met15Δ0; tlc1::NAT; rnh201::HYG*
yVP347	Survivor (*rnh201Δ*) to be transformed with pBL97 (EV) or pBL401 (RNH201)	*MATa; his3Δ1; leu2Δ0; ura3Δ0; met15Δ0; tlc1::NAT; rnh201::HYG*
ySM518-530	Survivor (*rnh201Δ*) to be transformed with pBL97 (EV) or pBL401 (RNH201)	*his3Δ1; leu2Δ0; ura3Δ0; met15Δ0; est2::HYG; rnh201::NAT*
ySM123	yDB05 passaged in liquid culture: 1L telomere length is of ~ 400 bp)	*MATa; his3Δ1; leu2Δ0; ura3Δ0; met15Δ0; bar1::KAN; tlc1::HIS3*
yCW450, 454, 458	Wild-type transformed with pBL906 (EV)	*MATa; his3Δ1; leu2Δ0; ura3Δ0; met15Δ0; osTIR1(F74G)-Leu2 + pBL906 (pRS416)*
yCW452, 456, 460	Wild-type transformed with pBL837 (RNase H1 cd)	*MATa; his3Δ1; leu2Δ0; ura3Δ0; met15Δ0; osTIR1(F74G)-Leu2 + pBL837 (pRS416 GAL-RNH1-(D193N)-HA)*
yCW466, 470, 640	Survivor transformed with pBL906 (EV)	*MATa; his3Δ1; leu2Δ0; ura3Δ0; met15Δ0; tlc1::NAT; osTIR1(F74G)-Leu2 + pBL906 (pRS416)*

yCW468, 472, 642	Survivor transformed with pBL837 (RNase H1 cd)	*MATa; his3Δ1; leu2Δ0; ura3Δ0; met15Δ0; tlc1::NAT; osTIR1(F74G)-Leu2 Leu2 + pBL837 (pRS416 GAL-RNH1-(D193N)-HA)*
yCW598-600	*sir2Δ* transformed with pBL906 (EV)	*MATa; his3Δ1; leu2Δ0; ura3Δ0; met15Δ0; sir2::KAN + pBL906 (pRS416)*
yCW604-606	*sas2Δ* transformed with pBL906 (EV)	*MATa; his3Δ1; leu2Δ0; ura3Δ0; met15Δ0; sas2::KAN + pBL906 (pRS416)*

Supplementary Table S2: Plasmids used in this study

Name	Description
pSM2	pRS316; *TLC1;* CEN; *URA3*
pBB39	pRS426; *pGPD-RNH1-HA;* 2μ; *URA3*
pBL97	pRS316; CEN; *URA3*
pBL211	pRS425; *pGal*; 2μ; *LEU2*
pBL352	pRS425; *pGal-RNH1-HA*; 2μ; *LEU2*
pBL189	pRS426; *pGPD*; 2μ; *URA3*
pBL401	pRS316; *RNH201*; CEN; *URA3*
pBL906	pRS416; *pGal;* CEN; *URA3*
pBL837	pRS416; *pGal-Rnh1(D193N)-HA;* CEN; *URA3*

Supplemental Table S3: Oligonucleotides used in this study

Name	Sequence	Purpose
oSM1	GAAGCGGATGGTAATGAGAC	Fw – 1L probe for Southern blot
oSM2	AGATGTATGATGCTGGGGAG	Rv – 1L probe for Southern blot; Junction PCR: 1L tel
oSM3	TCTTGATGTGTCTTCACAAG	Fw – 15L probe for Southern blot
oSM4	ATTCTCACCATCAAAGAAG	Rv – 15L probe for Southern blot
oSM5	CTATCTGCTTAGTCGAGGAGAAC	Fw – Junction PCR: Y' elements
oSM9	ATGCTGCCATGGATTCAACC	Rv – Junction PCR: 15L tel
oLK49	GGCTTGGAGGAGACGTACATG	Fw – RT-qPCR: 6 Y' tel
oLK50	CTCGCTGTCACTCCTTACCCG	Rv – RT-qPCR: 6 Y' tel
oLK57	GGGTAACGAGTGGGGAGGTAA	Fw – RT-qPCR: 15L tel

oLK58	CAACACTACCCTAATCTAACCCTGT	Rv – RT-qPCR: 15L tel
oBL207	CACCACACCCACACACCACACCCACA	Reverse transcription
oBL240	AAGAGGCATACCTCCGCCTATC	Rv – Reverse transcription; RT-qPCR: Tlc1
oBL241	TTGGTGTTGTATTCACAGCT	Fw – RT-qPCR: Tlc1
oBL292	CCCAGGTATTGCCGAAAGAATGC	Fw – RT-qPCR: Actin
oBL293	TTTGTTGGAAGGTAGTCAAAGAAGCC	Rv – RT-qPCR: Actin
oBL295	CGGTGGGTGAGTGGTAGTAAGTAGA	Fw – RT-qPCR: 1L tel
oBL296	ACCCTGTCCCATTCAACCATAC	Rv – RT-qPCR: 1L tel
oBL358	GCGGTACCAGGGTTAGATTAGGGCTG	Rv – Telo-PCR: 1L tel
oBL359	CGGGATCCGGGGGGGGGGGGGGGGG	Fw – Telo-PCR: oligo-dG
